# Multi-Omics Analysis and Comparison of the Developmental Characteristics of Muscle Fiber Types Between Huainan and Large White Pigs in Early Postnatal Period

**DOI:** 10.3390/biology14101409

**Published:** 2025-10-14

**Authors:** Jing Wang, Mingyang Jia, Hanbing Zhang, Yaping Guo, Qi Zhang, Xiangzhou Yan, Qingxia Lu, Sihuan Zhang, Baosong Xing

**Affiliations:** 1 Key Laboratory of Livestock and Poultry Breeding and Nutrition Regulation in Henan Province, Henan Pig Breeding Engineering Research Centre, Institute of Animal Husbandry, Henan Academy of Agricultural Sciences, Zhengzhou 450002, China; wangjing@hnagri.org.cn (J.W.); mingyangjia@hnagri.org.cn (M.J.); z13783826050@163.com (H.Z.); ypguo525@163.com (Y.G.); zhangqi0230@163.com (Q.Z.); xiangzhouyan@hnagri.org.cn (X.Y.); qxlu1982@126.com (Q.L.); 2 College of Veterinary Medicine, Henan Agricultural University, Zhengzhou 450046, China; 3 College of Animal Science and Technology, Anhui Agricultural University, Hefei 230036, China

**Keywords:** Huainan pig, Large White pig, muscle fiber type, transcriptomics, metabolomics

## Abstract

**Simple Summary:**

This study compared the characteristics of muscle fiber types of the longissimus dorsi muscle between the Huainan pigs and the Large White pigs at four postnatal stages (0, 7, 14, and 21 days). Using myosin ATPase staining, transcriptomics, and metabolomics analysis, we found that Huainan pigs had a higher proportion of oxidative muscle fibers, which support superior aerobic energy metabolism and contribute to tender, flavorful meat. Key genes such as *KLF4*, *NOS1*, *SH3KBP1*, and *TRARG1* showed higher expression in Huainan pigs, regulating muscle fiber type composition by enhancing mitochondrial function, promoting nitric oxide synthesis, and modulating nutrient metabolism. Metabolomics analysis revealed significantly higher levels of carnosine, citrulline, and glycerol-3-phosphate in Huainan muscle, which enhance energy supply, antioxidant capacity, and fatty acid oxidation to promote muscle fiber transformation. Notably, an “energy-sensing” regulatory loop involving *KLF4* and citrulline (mediated by the AMPK pathway) was identified in Huainan pigs, synergistically enhancing mitochondrial function and fiber differentiation. These findings elucidate how genetic background influences muscle development and meat quality, providing novel targets for breeding high-quality pork breeds.

**Abstract:**

To investigate the molecular mechanisms underlying muscle fiber development in different pig breeds and their impact on meat quality, this study collected the longissimus dorsi muscle of the indigenous Huainan pig and the commercial Large White pig at four developmental stages (0, 7, 14, and 21 days postnatal). Muscle fiber types were identified using myosin ATPase staining, while transcriptomics and non-targeted metabolomics were employed to analyze differences in gene expression and metabolite composition. The results showed that the Huainan pig had a higher proportion of oxidative muscle fibers, indicating superior aerobic metabolic capacity and meat quality. Transcriptome data identified 18 differentially expressed genes common to both pig breeds, including *KLF4*, *NOS1*, *SH3KBP1*, and *TRARG1*, which were upregulated in Huainan pigs to regulate muscle fiber type composition and meat quality by influencing mitochondrial function, nitric oxide synthesis, and glucose/lipid metabolism. Metabolomics analysis revealed significantly elevated levels of carnosine, citrulline, serine, and glycerol-3-phosphate in Huainan pigs, which are associated with metabolic pathways promoting muscle fiber transformation via enhancing energy supply, antioxidant capacity, and fatty acid oxidation. Notably, integrated transcriptome–metabolome analysis showed that oxidative metabolism genes (e.g., *KLF4*) and metabolites (e.g., citrulline) formed an AMPK-mediated ‘gene–metabolite’ loop in Huainan pigs, which synergistically promotes mitochondrial function and fiber differentiation. In summary, this study provides new insights into the molecular mechanisms underlying meat quality differences between pig breeds and offers a theoretical basis for the breeding and development of high-quality pork.

## 1. Introduction

With the improvement of living standards, consumers’ demand for pork quality continues to grow, and meat quality has become a core issue in animal science research and livestock production. As the main component of pork, skeletal muscle growth and development directly determine meat yield and quality [1]. Compared with Western commercial pig breeds, Chinese indigenous pig breeds generally have better meat quality [2,3]. For example, the Large White pig, a typical Western commercial breed widely used globally, has a fast growth rate and high lean meat percentage but has quality defects such as low intramuscular fat content and pale meat color [4]; on the other hand, the Huainan pig, a representative indigenous breed originating from the Huainan region of Henan Province is famous for its excellent quality characteristics such as bright-red meat color, fine muscle texture, and high intramuscular fat content [5]. However, the molecular mechanism underlying this breed-specific difference has not been fully elucidated.

As the basic functional unit of skeletal muscle, muscle fiber type is a key factor determining meat quality. According to metabolic characteristics, muscle fibers can be divided into oxidative types (type I, type IIa, relying on aerobic metabolism) and glycolytic types (type IIx, type IIb, relying on anaerobic glycolysis) [6]. Muscles with a high proportion of oxidative fibers usually have higher pH value, better water-holding capacity, tenderness, and flavor, while glycolytic fibers, although positively correlated with growth rate, can reduce meat quality [7]. Studies have confirmed that muscle fiber type composition is a key factor contributing to meat quality differences between Chinese and foreign pig breeds. Western commercial breeds are primarily dominated by type IIb glycolytic fibers, whereas Chinese indigenous breeds are dominated by type I oxidative fibers [8,9,10]. For example, Guo et al. found that Jinhua pigs (a Chinese indigenous breed) have a higher proportion of oxidative fibers and stronger oxidase activity, which directly correlates with increased intramuscular fat deposition and reduced drip loss [8].

The formation of muscle fiber types is regulated by the synergistic interaction of gene expression and metabolic networks. Existing studies have shown that some genes are involved in muscle fiber transformation, such as *MYH7* (myosin heavy chain 7), *MYH4* (myosin heavy chain 4), *KLF4* (KLF transcription factor 4), and *PGC-1α* (PPARG coactivator 1 alpha). Type I oxidative fibers highly express *MYH7*, rely on mitochondrial aerobic respiration, and have high activity of oxidases. Type II glycolytic fibers highly express *MYH4*, rely on anaerobic glycolysis, and have significantly increased lactate dehydrogenase activity [11]. *KLF4* promotes the formation of oxidative fibers by enhancing mitochondrial function [12]. *PGC-1α* drives type I fiber formation by regulating mitochondrial respiration and fatty acid oxidation [13]. At the metabolic level, metabolites such as carnosine and citrulline affect muscle fiber characteristics by regulating energy supply and antioxidant capacity [14,15]. However, studies on the dynamic expression of muscle fiber type-specific genes, the variation rules of key metabolites, and the “gene-metabolite” interaction network in the early developmental stages of Huainan pigs and Large White pigs are still insufficient, and are the core of analyzing the formation of breed-specific meat quality.

Studies found that the early postnatal period in pigs is the critical period for the formation of different types of muscle fibers [16]. Thus, this study used myosin adenosine triphosphatase (ATPase) staining, transcriptome sequencing, and metabolome sequencing methods to systematically analyze the characteristics of muscle fiber types, gene expression, and metabolism in the longissimus dorsi muscle of Huainan pigs and Large White pigs in the early postnatal period (0, 7, 14, and 21 days postnatal). Then we integrated multi-omics data to identify co-regulatory pathways and key gene–metabolite pairs that regulate muscle fiber development. The results of this study will provide new insight for understanding the molecular mechanism of muscle fiber type differences between the two breeds, and provide potential target genes and metabolic markers for high-quality pork breeding.

## 2. Materials and Methods

### 2.1. Preparation of Experimental Animals and Collection of Samples

The Huainan (HN) and Large White (LW) pigs used in this study were purebred individuals from Henan Xingrui Agricultural and Animal Husbandry Technology Co., Ltd. (Xinyang, China). All animals were reared at the Animal Experimental Base of Henan Academy of Agricultural Sciences under uniform conditions: temperature maintained at 28–32 °C (0–7 days) and 25–28 °C (8–21 days) and humidity at 60–70%; fed a commercial creep feed with crude protein ≥ 20%, crude fat ≥ 5%, and lysine ≥ 1.2%; and housed in individual pens with sterilized wood shavings. Feed and water were provided ad libitum.

In this study, the longissimus dorsi muscles of Huainan pigs and Large White pigs at four postnatal stages (0, 7, 14, and 21 days postnatal) were collected to investigate histomorphological differences and analyze transcriptomic and metabolomic profiles. Three biological replicates were included for each breed at each time point. All animals were housed under similar management conditions. Prior to sampling, piglets were fasted for 12 h and deprived of water for 3 h. Euthanasia was performed by intravenous injection of barbiturate. Longissimus dorsi muscle tissue samples were then collected. A portion of each sample (approximately 1 cm × 1 cm × 1.5 cm tissue block) was used for histological analysis and preserved in an environmentally friendly GD fixative (Solarbio, Beijing, China) for subsequent paraffin embedding and sectioning. The remaining tissue was divided into two parts for RNA extraction and metabolomic sequencing. The processing steps were as follows: blood vessels were removed from the samples, and the tissue was rinsed three times with Dulbecco’s Phosphate-Buffered Saline (DPBS) (Solarbio, Beijing, China) containing 1 × penicillin and streptomycin (100 U/mL penicillin and 100 μg/mL streptomycin) (Solarbio, Beijing, China). After thorough cleaning, the samples were placed in cryovials, rapidly frozen in liquid nitrogen, and then stored at −80 °C. Samples were subsequently shipped on dry ice to Hangzhou Lianchuan Biotechnology Co., Ltd. (Hangzhou, China) for transcriptomic and metabolomic sequencing.

### 2.2. ATPase Staining of Longissimus Dorsi Muscle and Muscle Fiber Cross-Sectional Area Analysis

The longissimus dorsi muscle samples fixed in GD solution (Solarbio, Beijing, China) were subjected to paraffin embedding following a standard procedure, including gradient ethanol dehydration, xylene clearing, and paraffin infiltration. Frozen sections were prepared for ATPase staining (pH = 4.45–4.47) (Solarbio, Beijing, China) [3]. After preincubation under both acidic and alkaline conditions, 100–200 μL of staining working solution was applied to each section, followed by incubation at 37 °C for 30–45 min. Muscle fiber types were identified based on ATPase activity at pH 4.45–4.47: type I fibers (dark brown, slow-twitch oxidative) and type II fibers (light brown, including fast-twitch oxidative-glycolytic type IIa and glycolytic type IIx/IIb). For metabolic classification, ‘oxidative muscle fibers’ include type I and type IIa fibers, while ‘glycolytic muscle fibers’ refer to type IIx/IIb fibers. Muscle fiber types were then observed under a light microscope (Olympus BX53, Tokyo, Japan) at a magnification of 200×. Muscle fiber cross-sectional area was measured by selecting and quantifying at least 100 individual muscle fibers from each muscle section using Image J software (version 1.52v). The data were collected from three animals per group at each developmental stage (0, 7, 14, and 21 days). Statistical significance was determined using a two-way ANOVA followed by Tukey’s post hoc test.

### 2.3. Analysis of Transcriptome Sequencing Data

#### 2.3.1. RNA Extraction, Library Construction, and Sequencing

Tissue samples were ground into powder using a mortar and pestle pre-chilled with liquid nitrogen, and total RNA was extracted using Trizol reagent (Invitrogen, Carlsbad, CA, USA). RNA purity and concentration were measured with a microvolume UV-visible spectrophotometer (Nanodrop One, Thermon Fisher Scientific, Waltham, MA, USA), while RNA integrity and concentration were assessed using an Agilent 2100 Bioanalyzer. All samples exhibited RIN values between 8.2 and 9.5, meeting the quality standard (RIN ≥ 7) for transcriptome sequencing.

For each sample, 5 μg of total RNA was used for library construction. Poly(A)-tailed mRNA was enriched using Oligo(dT) magnetic beads (Thermo Fisher Scientific, Waltham, MA, USA), and double-stranded cDNA was synthesized using dNTPs (Takara Bio, Kusatsu, Japan) as substrates with SuperScript III Reverse Transcriptase (Thermo Fisher Scientific). The resulting cDNA was subjected to end repair using the End Repair Mix (New England Biolabs, Ipswich, MA, USA), which catalyzes the removal of 3′ overhangs and filling of 5′ overhangs to generate blunt ends. A-tailing was performed by adding a single adenine residue to the 3′ end of the blunt cDNA using Klenow Fragment (3′→5′ exo^−^, New England Biolabs, Ipswich, MA, USA) and dATP (Takara Bio, Kusatsu, Japan). Adaptor ligation was conducted with *T4* DNA Ligase (New England Biolabs, Ipswich, MA, USA) and Illumina-compatible sequencing adaptors (Illumina, San Diego, CA, USA) to link the adaptors to the A-tailed cDNA fragments. Fragments of 370–420 bp were selected using 2% agarose gel electrophoresis (agarose from Biowest, Spain) with a DNA marker (Thermo Fisher Scientific, Waltham, MA, USA) for size reference; target bands were excised and purified using the QIAquick Gel Extraction Kit (Qiagen, Hilden, Germany). PCR amplification was performed in a 50 μL reaction system containing 25 μL of 2 × Phusion High-Fidelity PCR Master Mix (New England Biolabs, Ipswich, USA), 2 μL of each primer (10 μmol/L), 5 μL of purified cDNA fragments, and 16 μL of ddH_2_O. The PCR program was as follows: initial denaturation at 98 °C for 30 s; 12 cycles of 98 °C for 10 s, 60 °C for 30 s, and 72 °C for 30 s; final extension at 72 °C for 5 min.

Quality control for the libraries included the following: (1) concentration determination using a Qubit 4 Fluorometer (Thermo Fisher Scientific, Waltham, MA, USA) to ensure ≥2 nM; (2) size distribution analysis via Agilent 2100 Bioanalyzer (Agilent Technologies, Santa Clara, CA, USA) with the High Sensitivity DNA Kit to confirm the main peak within 370–420 bp; (3) sequencing of a small portion (100,000 reads) on the Illumina platform to check for adapter contamination and low-quality bases (Q20 ≥ 97%, Q30 ≥ 93%). Libraries that passed quality control were sequenced on the Illumina HiSeq 2000 platform using high-throughput sequencing. Reads were aligned to the Sus scrofa reference genome (version Sscrofa11.1, https://www.ncbi.nlm.nih.gov/assembly/GCF_000003025.6/, accessed on 8 September 2024) using Hisat2 (version 2.2.1) software. The alignment process included the following: (1) building a genome index based on the reference sequence; (2) aligning paired-end clean reads to the reference genome with default parameters (maximum insert size = 500 bp, mismatch penalty = 6); (3) filtering aligned reads to retain those with mapping quality ≥ 20 for subsequent analysis.

For sequencing data quality control, the overall error rate was calculated as the ratio of the total number of incorrect base calls to the total number of base calls across all reads. An error was defined as a base call with a Phred quality score < 20 (corresponding to a base-calling accuracy < 99%), as determined using FastQC software (version 0.11.9). This threshold was selected based on standard criteria for ensuring reliable downstream analysis of sequencing data.

#### 2.3.2. Screening and Functional Annotation of Differentially Expressed Genes

Gene transcript abundance was quantified as FPKM (Fragments per kilobase of exon model per million mapped fragments) to represent the level of gene expression. Differential expression analysis was performed using the negative binomial distribution model implemented in DESeq2 (version 1.20.0). Genes with a *q*-value < 0.05 (*q*-value: adjusted *p*-value for multiple testing using the Benjamini–Hochberg method) and |log_2_foldchange| ≥ 1 were considered differentially expressed genes (DEGs). The clusterProfiler package (version 3.8.1) was used to perform Gene Ontology (GO) enrichment analysis (http://www.geneontology.org, accessed on 10 September 2024) and Kyoto Encyclopedia of Genes and Genomes (KEGG) pathway enrichment analysis (http://www.kegg.jp, accessed on 10 September 2024) for the DEG sets. A threshold of *p* < 0.05 was considered statistically significant, while *p* < 0.01 was regarded as highly significant enrichment.

### 2.4. Analysis of Metabolome Sequencing Data

After snap-freezing in liquid nitrogen, longissimus dorsi muscle samples were sent to Lianchuan Biotechnology Co., Ltd. for untargeted metabolomic analysis using LC-MS/MS. Metabolite detection was performed on a UHPLC–HRMS (Ultra-High-Performance Liquid Chromatography–High-Resolution Mass Spectrometry) platform consisting of a Thermo Ultimate 3000 UHPLC system coupled with a Q Exactive HF-X mass spectrometer (Thermo Fisher Scientific, Waltham, MA, USA) under both positive and negative ionization modes to maximize metabolite coverage. The UHPLC separation was conducted using an ACQUITY UPLC BEH C18 column (2.1 mm × 100 mm, 1.7 μm; Waters, Milford, MA, USA) with a column temperature of 40 °C. The mobile phase for the positive ionization mode consisted of (A) 0.1% formic acid in water and (B) 0.1% formic acid in acetonitrile, while for the negative ionization mode, it consisted of (A) 5 mM ammonium acetate in water (pH 9.0) and (B) acetonitrile. The gradient elution program was as follows: 0–1 min, 5% B; 1–9 min, 5–95% B; 9–12 min, 95% B; 12–12.1 min, 95–5% B; 12.1–15 min, 5% B, with a flow rate of 0.3 mL/min and an injection volume of 2 μL. For mass spectrometry, the scanning range was set to *m*/*z* 70–1050, and the resolution was 60,000 for full scan and 15,000 for MS/MS scan.

Raw data were subjected to peak detection, alignment, de-noising, and quantification, with low-quality peaks removed. Quality control (QC) samples were prepared by pooling equal volumes (10 μL) of all experimental samples, ensuring they contained a representative mixture of metabolites from all groups. QC samples were injected at regular intervals (every 10 samples) throughout the sequencing run to evaluate instrument stability and data reliability by monitoring the relative standard deviation (RSD) of metabolite intensities (RSD < 30% for >80% of metabolites was considered acceptable).

After normalization, principal component analysis (PCA) was conducted using the prcomp function in R software (version 4.2.1) to assess the overall distribution of samples and intergroup variation. The analysis was performed on the normalized metabolite intensity data matrix, with autoscaling (mean centering and scaling to unit variance) applied to the data prior to PCA to eliminate the influence of different magnitude ranges among metabolites. Principal components (PCs) were extracted based on eigenvalue decomposition, and the first two principal components (PC1 and PC2) were used to generate scatter plots for visualizing sample clustering and variation. Normalization was performed using the probabilistic quotient normalization (PQN) method, which corrects for systematic variations by scaling each sample to a reference spectrum derived from the median of all samples. The normalized variables included the peak intensities of all detected metabolites to ensure comparability between samples.

Subsequently, orthogonal partial least squares discriminant analysis (OPLS-DA) was employed using the ropls package (version 1.26.0) in R software (version 4.2.1) to further identify metabolic differences between breeds and developmental stages. The analysis was performed on the normalized metabolite intensity matrix, with data preprocessing including mean centering and Pareto scaling (scaling to the square root of variance) to balance the influence of high- and low-intensity metabolites. A 7-fold cross-validation was used to determine the optimal number of latent variables, and the model’s goodness-of-fit was evaluated by R^2^Y (cumulative variance explained in the response variable) and Q^2^ (cumulative cross-validated variance explained), with R^2^Y > 0.7 and Q^2^ > 0.5 indicating a stable and reliable model. Permutation tests (1000 permutations) were conducted to assess model overfitting, ensuring the observed differences were not due to random variation. Differential metabolites were identified based on the criteria of VIP (variable importance in projection) > 1.0 and *p* < 0.05. By intersecting the differential metabolites of each breed across its four developmental stages (0, 7, 14, and 21 days), the common differential metabolites shared across the four developmental stages for each breed were identified. A further intersection was performed to obtain the differential metabolites shared by both breeds across the four developmental stages. Identified differential metabolites were structurally annotated by matching their accurate mass (mass error < 10 ppm) and MS/MS fragmentation patterns with the Human Metabolome Database (HMDB, https://hmdb.ca/, accessed on 12 September 2024), METLIN (https://metlin.scripps.edu/, accessed on 12 September 2024), and LIPID Maps (https://www.lipidmaps.org/, accessed on 12 September 2024) databases. For pathway enrichment analysis, the KEGG (http://www.kegg.jp, accessed on 12 September 2024) and MetaboAnalyst (https://www.metaboanalyst.ca, accessed on 12 September 2024) databases were used, with hypergeometric tests applied to calculate the significance of enrichment (*p* < 0.05). Key metabolic pathways associated with muscle fiber type, energy metabolism, lipid metabolism, and meat flavor were screened based on the number of differential metabolites involved and their functional relevance, with pathways containing ≥ 3 differential metabolites considered biologically meaningful.

### 2.5. qRT-PCR Validates Accuracy of Sequencing Data

Total RNA was extracted from the same tissue samples used for transcriptome library construction (as described in Section 2.3.1) using Trizol reagent. First-strand cDNA was synthesized using reverse transcriptase and oligo (dT) primers according to the instructions of the MonScript™ RTIII All-in-One Mix with dsDNase kit (Monadbiotech, Wuhan, China). Quantitative real-time PCR (qRT-PCR) was performed using the 2× Q3 SYBR qPCR Master Mix (TOLOBIO, Shanghai, China) and the CFX Connect Real-Time PCR Detection System (Bio-Rad, Berkeley, CA, USA). Primers were designed based on sequences obtained from the NCBI database (Table 1) and synthesized by TsingKe Biotechnology Co., Ltd. (Wuhan, China). *GAPDH* was used as the internal reference gene. The total reaction volume for qRT-PCR was 20 μL, consisting of 10 μL of premix, 8.2 μL of ddH_2_O, 1 μL of cDNA template, and 0.4 μL each of forward and reverse primers (10 μmol/L). The qRT-PCR results were normalized using the 2^−ΔΔCt^ method with *GAPDH* as the internal reference gene.

## 3. Results

### 3.1. Morphological Changes in the Longissimus Dorsi Muscle Fibers at Different Developmental Stages

**Figure 1:**
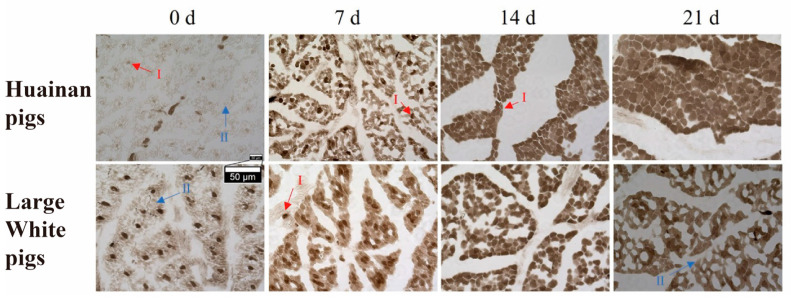
Myosin ATPase staining of longissimus dorsi muscle of Huainan pigs and Large White pigs at four postnatal stages (0, 7, 14, and 21 days). I, type I fibers (dark brown); II, type II fibers (light brown); n = 3 biological replicates per group.

To investigate the characteristics and composition of muscle fiber types at different developmental stages in Huainan pigs and Large White pigs, myosin ATPase staining (pH 4.45–4.47) was performed to identify muscle fiber types in both breeds. Two fiber types were identified across the four stages (0, 7, 14, and 21 days postnatal) in both Huainan pigs and Large White pigs: type I fibers (dark brown) and type II fibers (light brown) (Figure 1). At birth (day 0), Huainan pigs exhibited a higher proportion of type II fibers, whereas Large White pigs had more type I fibers. At day 21, the proportion of type I was higher in Huainan pigs compared to Large White pigs. Statistical analysis of muscle fiber area revealed a significant difference between the two breeds at day 21 (Table 2), with the muscle fiber area of Huainan pigs being 10.55% larger than that of Large White pigs.

### 3.2. Dynamic Transcriptome Changes in Longissimus Dorsi Muscle at Four Developmental Stages in Huainan and Large White Pigs

#### 3.2.1. Sequencing Data Quality Control

Transcriptome sequencing was performed on longissimus dorsi muscle of Large White pigs and Huainan pigs at four developmental stages. Large White and Huainan pigs generated 44.38 million and 39.54 million clean reads per sample, respectively. The overall sequencing error rates were below 0.03%, with Q20 scores exceeding 99.95% and Q30 scores above 97.02%, and the average mapping rate was >94% across all samples, indicating high-quality sequencing data (Supplementary Table S1).

#### 3.2.2. Differential Transcriptome Analysis Across Four Developmental Stages

**Figure 2:**
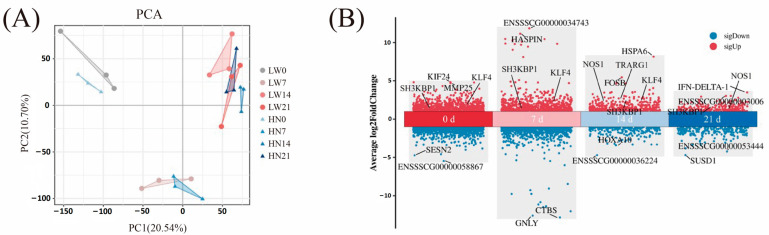
Transcriptome sequencing data analysis. (**A**) PCA. (**B**) Differentially expressed genes between Huainan pigs and Large White pigs at four postnatal stages (0, 7, 14 and 21 days). Red and blue dots represent genes with higher and lower expression in Huainan pigs, respectively. Each dot represents a gene. Key genes mentioned in the text (e.g., *KLF4*, *NOS1*, *SH3KBP1*, *TRARG1*) are highlighted. HN represents Huainan pigs; LW represents Large White pigs.

The PCA results showed that the samples of day 0 and day 7 clustered separately from those of day 14 and day 21, indicating distinct gene expression profiles between early (day 0 and day 7) and later (day 14–21) developmental stages (Figure 2A). This separation was consistent with the dynamic transcriptional changes revealed by DEGs analysis, where more DEGs were identified between day 7 vs. day 0 and day 14 vs. day 7 compared to day 21 vs. day 14 (Supplementary Figure S1A, Supplementary Table S2). In Huainan pigs, 2777 DEGs were identified between days 0 and 7, 3405 between days 7 and 14, and only 147 between days 14 and 21. Similarly, Large White pigs showed 2982 DEGs between days 0 and 7, 3261 between days 7 and 14, and 105 between days 14 and 21 (Supplementary Table S2). These results suggest that the period from birth to day 7 is critical for muscle development, whereas muscle development stabilizes between days 14 and 21 (Supplementary Figure S2A).

#### 3.2.3. Differential Analysis of Transcriptome Between Huainan Pigs and Large White Pigs

Regarding breed differences, we conducted PCA and calculated DEGs for each age group. The PCA results showed that Huainan and Large White pigs formed distinct clusters at day 0, day 14, and day 21, indicating significant inter-breed transcriptional divergence at these stages (Supplementary Figure S2). The DEGs analysis for each age group further confirmed clear breed separation at these stages (Figure 2A, Supplementary Table S3). The numbers of DEGs in Huainan and Large White pigs at days 0, 7, 14, and 21 are 1737, 181, 426, and 131, respectively (Supplementary Table S3). The decreasing number of DEGs over time further indicates that transcriptomic differences between the two breeds are more pronounced during early postnatal development.

#### 3.2.4. Expression Analysis of Different Muscle Fiber Type Marker Genes

**Figure 3:**
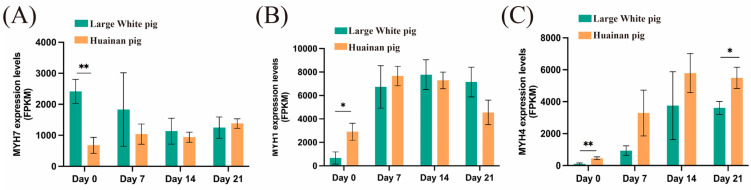
Expression levels of different muscle fiber type marker genes. (**A**) MYH7; (**B**,**C**) MYH1 and MYH4. Data are presented as mean ± SEM (n = 3 biological replicates per group). Statistical significance was determined using two-way ANOVA followed by Tukey’s post hoc test; * and ** represent *p* < 0.05 and *p* < 0.01, respectively.

We analyzed the expression levels of marker genes associated with different muscle fiber types. At day 0, the oxidative muscle fiber marker gene *MYH7* [11] expressed significantly higher in Large White pigs compared to Huainan pigs (Figure 3A and Supplementary Table S3), while the glycolytic muscle fiber marker genes *MYH1* and *MYH4* [11] showed significantly higher expression levels in Huainan pigs compared to Large White pigs (Figure 3B,C and Supplementary Table S3). These expression patterns of muscle fiber type-specific marker genes are consistent with the muscle fiber phenotypes observed through ATPase staining at day 0. At day 21, the expression level of *MYH4* in Huainan pigs remained significantly higher compared to Large White pigs (Figure 3C), suggested that Huainan pigs had stronger development activity of glycolytic muscle fiber than Large White pigs at this stage.

#### 3.2.5. Screening of Key Genes for Muscle Fiber Development

To identify potential key regulatory genes, we performed a Venn analysis of DEGs across four developmental stages (Supplementary Table S4). A total of 18 core genes were identified that were consistently differentially expressed at all stages (Supplementary Figure S1B; Supplementary Table S5). Previous studies have shown that these genes play important roles in muscle fiber type transformation and meat quality formation. The Fos proto-oncogene, AP-1 transcription factor subunit (FOS) is a critical transcription factor involved in the regulation of muscle cell proliferation and differentiation [17]. The thyroid hormone responsive (THRSP) and diacylglycerol O-acyltransferase 2 (DGAT2) are closely associated with intramuscular fat synthesis and deposition, which may influence meat tenderness and flavor [18,19]. The activating transcription factor 3 (*ATF3*), a stress-related transcription factor, regulates the activation of skeletal muscle stem cells by modulating H2B expression—specifically, it prevents the precocious activation of these stem cells to maintain their regenerative potential [20]. The transcription factor KLF4 promotes the differentiation and maintenance of oxidative muscle fibers by activating genes related to mitochondrial biogenesis (such as PGC-1α) [21,22]. The nitric oxide synthase 1 (NOS1) activates mitochondrial uncoupling proteins (UCPs), enhances oxidation efficiency, promotes angiogenesis, and meets the aerobic metabolism needs of oxidative muscle fibers [23]. The SH3 domain containing kinase-binding protein 1 (SH3KBP1) is mainly highly expressed in type I oxidative muscle fibers, and indirectly supports the metabolic characteristics of oxidative muscle fibers by regulating calcium signal transmission and enhancing mitochondrial oxidative phosphorylation efficiency [24]. The trafficking regulator of GLUT4 1 (TRARG1) is highly expressed in oxidative muscle fibers, and meets their high energy demands by enhancing glucose oxidative metabolism, thereby indirectly maintaining the phenotype of oxidative fibers [25]. The salt-inducible kinase 1 (SIK1) can promote the expression of genes related to mitochondrial oxidative metabolism and enhance the activity of the mitochondrial respiratory chain [26].

#### 3.2.6. Validation of Transcriptome Sequencing Results by qRT-PCR

**Figure 4:**
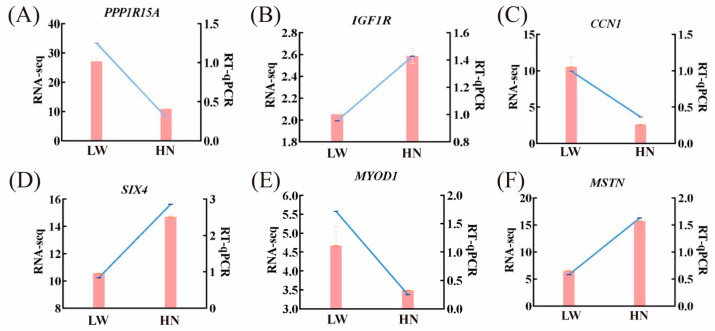
qRT-PCR validation of differentially expressed genes identified by RNA-seq. (**A**–**F**) respectively show qRT-PCR and RNA-seq validation of *PPPR11A*, *IGF1R*, *CCN1*, *SIX1*, *MYOD1*, and *MSTN* genes. Pink bars represent relative mRNA levels detected by qRT-PCR; blue lines represent FPKM values from RNA-seq data (transcriptome sequencing of 21-day-old longissimus dorsi muscle). Data are presented as mean ± SEM (n = 3 biological replicates per group). HN represented Huainan pigs; LW represented Large White pigs.

To validate the accuracy of the RNA-Seq data, six differentially expressed genes were randomly selected for qRT-PCR analysis. The qRT-PCR results showed that the expression patterns of these six genes were consistent with the RNA-Seq results (Figure 4).

#### 3.2.7. Functional Enrichment Analysis of Differentially Expressed Genes

**Figure 5:**
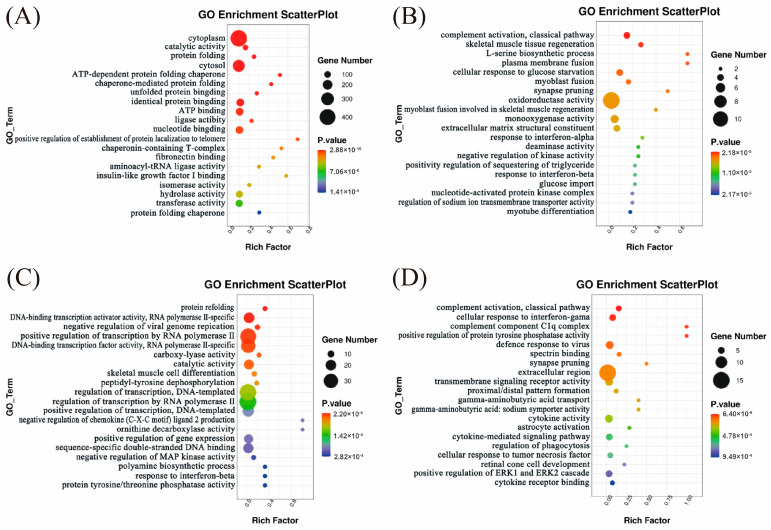
GO enrichment analysis of DEGs between Huainan pigs and Large White pigs at Day 0 (**A**), Day 7 (**B**), Day 14 (**C**), and Day 21 (**D**). Rich factor represents the proportion of DEGs in a certain GO term to the total number of genes in that GO term. The size of the circles in this figure represents the approximate range of the number of genes, not the specific number. For the specific number of genes in each pathway, please refer to Tables S6–S9.

To further investigate the biological functions of DEGs at various developmental stages, we performed GO functional annotation and KEGG pathway enrichment analyses on the DEGs identified between Huainan and Large White pigs at each developmental stage. GO enrichment analysis revealed that the DEGs between Huainan and Large White pigs at day 0 enriched in “ATP-dependent protein folding” and “fibronectin binding” pathways, which are closely associated with the early maturation of oxidative type I fibers. ATP-dependent chaperones support the correct folding of myofibrillar proteins (e.g., *MYH7*) in slow-twitch fibers, while fibronectin-mediated adhesion promotes the differentiation of myoblasts into oxidative fiber lineages—consistent with the higher proportion of type I fibers in Large White pigs at this stage (Figure 5A and Supplementary Table S6). At day 7, DEGs were significantly enriched in processes associated with skeletal muscle development and regeneration, including skeletal muscle tissue regeneration, plasma membrane fusion, myoblast fusion, and myotube differentiation, suggesting active muscle fiber development and significant differences between the two breeds during this stage (Figure 5B and Supplementary Table S7). At day 14, DEGs were primarily involved in skeletal muscle cell differentiation and protein refolding, indicating a transition toward muscle maturation accompanied by dynamic regulation of protein processing (Figure 5C and Supplementary Table S8). At day 21, DEGs were enriched in pathways related to neural signaling, synapse organization, signal transduction, and cytoskeleton regulation (Figure 5D and Supplementary Table S9). This suggests that neural regulation and signaling pathways may jointly contribute to muscle fiber development and the formation of meat quality traits at this stage.

The KEGG analysis results showed that at day 0, the DEGs were mainly enriched in pathways related to energy metabolism, such as metabolic pathways, glutathione metabolism, and the AMPK signaling pathway (Supplementary Figure S3A and Supplementary Table S10). The AMPK pathway can activate *PGC-1α* to promote mitochondrial biogenesis, enhance oxidative metabolism, and drive the conversion of muscle fibers to oxidative types [10]. The metabolic pathway provides material and energy foundations for muscle fiber type conversion. At day 7, significant enrichment was observed in the PPAR signaling pathway and adipocytokine signaling pathway, indicating notable differences between the two breeds in lipid metabolism and adipocyte function regulation at this stage (Supplementary Figure S3B and Supplementary Table S11). The PPAR and insulin signaling pathways are enriched. Members of the PPAR family can regulate muscle fiber types; for example, *PPARδ* (peroxisome proliferator activated receptor δ) activation promotes the differentiation of oxidative muscle fibers and enhances mitochondrial function, while the insulin pathway can affect the energy supply mode of muscle fibers [27]. At day 14, DEGs were primarily involved in the AMPK and MAPK signaling pathways as well as fatty acid biosynthesis, which are closely associated with energy metabolism and lipogenesis (Supplementary Figure S3C and Supplementary Table S12). This suggests that this period may represent a key phase for intramuscular fat deposition and related metabolic activity. The skeletal muscle cell differentiation pathway directly corresponds to the processes of myocyte differentiation and muscle fiber type determination, involving the regulation of key transcription factors such as myogenic differentiation (*MyoD*) and myogenic factor 5 (*Myf5*). At day 21, DEGs were enriched in amino acid and vitamin metabolism pathways (such as arginine biosynthesis and retinol metabolism), as well as cell signaling and developmental regulation pathways (including the apelin signaling pathway and cell adhesion molecules) (Supplementary Figure S3D and Supplementary Table S13). These findings suggest that significant differences in immune function, cell structure regulation, and muscle tissue remodeling still exist between the two breeds at this later stage. The apelin signaling pathway can regulate muscle energy metabolism and angiogenesis, indirectly affecting the oxidative capacity and type conversion of muscle fibers [28]. Although the NOD-like receptor signaling pathway is mainly involved in immunity, the inflammatory microenvironment can affect the metabolic phenotype of muscle fibers (e.g., chronic inflammation may inhibit the maintenance of oxidative fibers) [29]. In addition, pathways related to lipid deposition are enriched at all stages, such as fatty acid biosynthesis, adipocytokine signaling pathway, and PPAR signaling pathway.

### 3.3. Dynamic Metabolome Changes in Longissimus Dorsi Muscle at Four Developmental Stages in Huainan and Large White Pigs

#### 3.3.1. Statistical Analysis of Differential Metabolites

**Figure 6:**
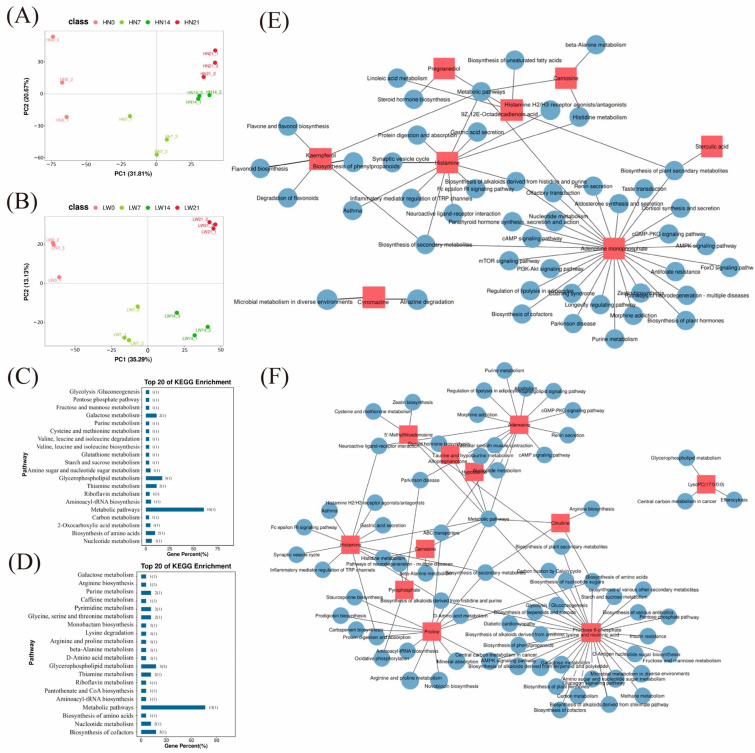
Metabolomics data analysis. PCA of metabolomic data in Huainan pigs (**A**) and Large White pigs (**B**). KEGG enrichment analysis of differential metabolites in Huainan pigs (**C**) and Large White pigs (**D**). Network diagram of the regulatory relationship between differential metabolites and KEGG pathways in Huainan pigs (**E**) and Large White pigs (**F**). HN represents Huainan pigs; LW represents Large White pigs.

To further explore the metabolic characteristics associated with different muscle fiber types, we performed untargeted metabolomic profiling of longissimus dorsi muscle samples collected from Huainan and Large White pigs at 0, 7, 14, and 21 days of age, respectively. A total of 595 secondary metabolites were identified after quality control filtering. OPLS-DA revealed clear metabolic distinctions between the two pig breeds at each developmental stage, indicating appropriate sample grouping and high data reliability (Figure 6A,B). Using VIP > 1 and *p* < 0.05 as the screening criteria, a total of 127 differential metabolites were identified across the four developmental stages (HN0 vs. HN7 vs. HN14 vs. HN21) in Huainan pigs (Supplementary Table S14). These metabolites mainly included organic acids and their derivatives, lipids and lipid-like molecules, aromatic compounds, and heterocyclic compounds. In contrast, 147 differential metabolites were identified in Large White pigs across the four developmental stages (LW0 vs. LW 7 vs. LW 14 vs. LW 21), primarily involving fatty acids, glycerophospholipids, organic nitrogen compounds, and organic oxygen compounds (Supplementary Table S14).

And there were 63 shared differential metabolites between these two breeds across the four developmental stages (LW0 vs. LW 7 vs. LW 14 vs. LW 21 vs. HN0 vs. HN 7 vs. HN 14 vs. HN 21) (Supplementary Table S14). These shared metabolites cover various chemical categories, including glycerol 3-phosphate, which is closely related to energy metabolism [30]; carnosine and hypotaurine with antioxidant functions [31,32]; and carnitine substances involved in lipid metabolism (such as butyrylcarnitine, 3-hydroxybutyrylcarnitine, and malonylcarnitine) [33]. Functional enrichment analysis indicated that these shared differential metabolites are mainly involved in pathways such as the AMPK signaling pathway, glycerophospholipid metabolism, β-alanine metabolism, and fatty acid oxidation. Among them, the levels of carnosine, glycerol 3-phosphate, etc., in Huainan pigs were significantly higher than those in Large White pigs, which echoed the high-expression pattern of oxidative metabolism-related genes (e.g., *KLF4*) in transcriptome analysis. This suggests that these metabolites may participate in the differentiation process of muscle fiber types by synergistically regulating mitochondrial function and energy metabolism, thereby affecting meat quality-related traits.

#### 3.3.2. Functional Enrichment Analysis of Differential Metabolites

The KEGG pathway enrichment analysis of the differential metabolites revealed that in Huainan pigs (HN0 vs. HN 7 vs. HN 14 vs. HN 21), the metabolites were enriched in pathways closely related to energy metabolism, including the pentose phosphate pathway, galactose metabolism, glutathione metabolism, and glycerophospholipid metabolism (Figure 6C). In Large White pigs (LW0 vs. LW 7 vs. LW 14 vs. LW 21), differential metabolites were predominantly enriched in signaling pathways including Ras, cGMP-PKG, and calcium signaling (Figure 6D). These findings indicate distinct metabolic profiles between muscle fiber types, providing a basis for identifying key metabolites that regulate fiber-type transition and meat quality formation. KEGG pathway enrichment analysis of the 63 differential metabolites shared between the two breeds across four developmental stages did not identify any statistically significant pathways (*p* > 0.05), indicating that their conserved roles in muscle development may involve scattered or context-dependent regulatory mechanisms rather than concentrated pathway activation.

To identify key metabolites involved in the regulation of muscle fiber type, we constructed a regulatory network diagram based on the top 30 differentially expressed metabolites and their associated KEGG pathways in Huainan pigs (Figure 6E) and Large White pigs (Figure 6F). Among the differentially expressed metabolites in Huainan pigs, histamine plays key role in the histamine H2 receptor antagonists/agonists pathway, which can regulate metabolic processes in muscle [34] (Figure 6E). The cGMP-PKG signaling pathway can regulate calcium signaling, mitochondrial biogenesis, and the expression of enzymes related to oxidative metabolism—all of which are crucial for the differentiation and function of oxidative muscle fibers [35] (Figure 6E). In Large White pigs, the metabolites kaempferol, sterculic acid, and carnosine synergistically regulate muscle fiber types through different energy metabolic pathways (Figure 6F). Kaempferol promotes mitochondrial biogenesis by activating PGC-1α signaling pathway [36]. Sterculic acid regulates energy metabolism by switching the utilization of metabolic substrates to fatty acid oxidation by inhibiting SCD1 [37]. Carnosine ensures continuous energy output primarily by buffering intracellular pH during glycolysis [38]. Taken together, these metabolites regulate the metabolic profile of muscle by regulating oxidative capacity, lipid utilization, and glycolytic stability.

## 4. Discussion

Until now, some studies have explored the metabolome differences in muscle among different pig breeds, but have mainly focused on the effects of the metabolome on pork flavor [3]. In this study, we analyzed the muscle fiber types, transcriptome and metabolome dynamics of longissimus dorsi muscle in Huainan pigs and Large White pigs in order to uncover the association of muscle fiber type with gene expression and tissue metabolism. A total of 18 core genes with differential expressions in both breeds were identified, among which 8 genes were highly expressed in Huainan pigs, which may affect the proportion of oxidative muscle fibers and meat quality by regulating mitochondrial function, oxidative metabolism and calcium homeostasis, and glucose–lipid metabolism. Meanwhile, the differential metabolites and their enriched pathways at different stages were analyzed, revealing the differences in muscle fiber development and metabolic characteristics between the two breeds. The results not only provide new insights into understanding the molecular mechanisms of pig muscle fiber type transformation and meat quality formation, but also offer a theoretical basis for the protection, development, and pork quality improvement of indigenous Huainan pig.

### 4.1. Difference Analysis and Significance of Muscle Fiber Types

The ATPase staining clearly revealed the dynamic changes in muscle fiber type composition in the longissimus dorsi muscle of Huainan and Large White pigs during postnatal development, as well as the breed-specific differences. As age increased, the proportion of type I fibers gradually rose in both breeds, suggesting that oxidative muscle fibers progressively became dominant during early piglet development. This shift may be closely related to increased growth rate, enhanced physical activity demands, and changes in metabolic patterns [39].

Notably, at day 21, the proportion of type I muscle fibers in Huainan pigs was significantly higher compared to Large White pigs. Studies have shown that indigenous breeds such as Ningxiang pigs and Bama Xiang pigs also tend to have a higher proportion of oxidative muscle fibers, which correlates with their slower growth rates and premium meat quality [3,10]. In contrast, Large White pigs exhibit a higher proportion of type II fibers, which may favor faster growth but is relatively disadvantageous for meat quality improvement [40]. Interestingly, at day 21, the muscle fiber cross-sectional area of Huainan pigs was 10.55% higher than that of Large White pigs. In general, type II muscle fibers have a larger diameter and cross-sectional area than type I muscle fibers, which allows them to contribute to muscle hypertrophy [41]. However, the above conclusions are usually drawn by comparing type I and type II muscle fibers of the same breed/individual [42]. The reason for the above result in this study may be that the diameter of the relatively mature type I muscle fibers in Huainan pigs is larger than that of the type II muscle fibers in Large White pigs at the initial stage of development due to genetic differences between breeds.

### 4.2. Regulation of Co-Differentially Expressed Genes

Transcriptome analysis identified 18 differentially expressed genes common to both breeds across four developmental stages. Eight genes (including *KLF4*, *NOS1*, MX Dynamin Like GTPase 1 (*MX1*), *SH3KBP1*, *TRARG1*, solute carrier family 6 member 13 (*SLC6A13*), *THRSP*, and ENSSSCG00000061569) showed significantly higher mRNA levels in the longissimus dorsi muscle of Huainan pigs, potentially contributing to their increased oxidative fiber proportion and improved meat quality. Notably, the transcription factor KLF4 enhances aerobic metabolism by promoting mitochondrial function and fatty acid oxidation, thereby maintaining oxidative fiber characteristics [43,44]. NOS1 is expressed in skeletal muscle and regulates nitric oxide (NO) levels. NO, as a signaling molecule regulated by lactate release, coordinates vasodilation by modulating mitochondrial function [45]. SH3KBP1 is an adaptor protein highly expressed in type I muscle fibers, it maintains the structural integrity of the sarcoplasmic reticulum, stabilizes mitochondria–endoplasmic reticulum interactions, and regulates Ca^2+^ homeostasis, thereby preferentially supporting the metabolic and contractile functions of oxidative muscle fibers [24]. TRARG1 is considered a key regulator of glucose transport in muscle cells, involved in GLUT4 translocation and insulin sensitivity. Its high expression promotes glucose oxidative metabolism, supporting the energy demands of oxidative muscle fibers [46,47]. THRSP is a known regulator of lipid metabolism, playing a critical role in fat synthesis and closely associated with intramuscular fat content and marbling in meat quality [48]. In summary, among the 18 common differentially expressed genes identified by transcriptome analysis, the 8 genes (such as *KLF4* and *NOS1*) with significantly higher expression levels in the longissimus dorsi muscle of Huainan pigs may collectively promote the increase in the proportion of oxidative muscle fibers and the improvement of meat quality by regulating biological processes such as mitochondrial function, oxidative metabolism, calcium homeostasis, and glucose–lipid metabolism.

### 4.3. Changes in Metabolites and Their Relationship with Muscle Fiber Types

We performed metabolomic analyses on the longissimus dorsi muscle of Huainan and Large White pigs at four developmental stages: 0, 7, 14, and 21 days after birth. A total of 11 metabolites were found to have significantly higher levels in Huainan pigs, which were predominantly enriched in pathways related to energy metabolism. They support oxidative fiber development by activating oxidative metabolism pathways (e.g., AMPK), maintaining mitochondrial membrane integrity, regulating sarcoplasmic reticulum Ca^2+^ homeostasis, or generating lipid signaling molecules. For wxample, carnosine, a β-alanine-histidine diptide abundant in skeletal muscle, exhibits antioxidant and anti-fatigue properties [48,49]. Glycerol 3-phosphate serves as a metabolic hub linking glycolysis and phospholipid synthesis, regulating myocyte membrane composition to maintain calcium signaling and contractile function [30]. Enhanced glycerophospholipid synthesis promotes the transition of glycolytic muscle fibers to oxidative muscle fibers by stabilizing mitochondrial function and Ca^2+^ handling [50]. MTA not only participates in polyamine synthesis and antioxidant responses but also plays a positive role in skeletal muscle regeneration and homeostasis maintenance [51]. Serine, an important substrate for one-carbon metabolism and glutathione (GSH) synthesis, contributes to the maintenance of mitochondrial redox homeostasis and supports the high energy metabolic capacity of oxidative muscle fibers [52]. Citrulline, a key intermediate in glutamine metabolism, promotes nitric oxide production and arginine regeneration, thereby enhancing mitochondrial function [53].

In addition, we found that the metabolic characteristics of longissimus dorsi in the early postnatal period (0–7 days of age) were closely related to the muscle fiber types in the later period (21 days of age). In this study, the key metabolites of oxidative metabolism such as creatine and citrulline in muscle of Huainan pigs at day 7 were significantly higher compared to Large White pigs, while the glycolytic metabolite lactic acid in Large White pigs was significantly higher compared to Huainan pigs. Correspondingly, the proportion of oxidized muscle fibers in Huainan pigs was significantly higher than that in Large White pigs at day 21. Studies have found that early metabolic reprogramming can affect the proportion of different types of muscle fibers at later stages by regulating key cellular signaling pathways, promoting mitochondrial production, and triggering long-term epigenetic modifications [54]. For example, citrulline can activate AMPK/peroxisome proliferator activated receptor alpha (PPARα) pathway [14]. The AMPK pathway is a key energy sensor that promotes mitochondrial biogenesis via PGC-1a. The balance between mitochondrial division and fusion during the neonatal period sets the stage for the generation of different types of muscle fibers [55]. The accumulation of these metabolites from 0 to 7 days continued to provide support for subsequent myofiber development. By day 21, the sum of early metabolic and environmental inputs had directed the developmental trajectory of the muscle toward a specific phenotype. In summary, the higher proportion of oxidative muscle fibers in Huainan pigs is reflected not only at the histological level but is also closely associated with their distinct metabolite spectra.

### 4.4. Correlative Analysis of Transcriptome and Metabolome

We conducted integrated transcriptomic and metabolomic analysis to reveal synergistic regulatory mechanisms between gene expression and metabolic phenotypes. Specifically, in Huainan pigs, multiple key “gene–metabolite” regulatory loops contribute to their high proportion of oxidative muscle fibers. For example, the KLF4 promotes the expression of genes related to mitochondrial biogenesis (e.g., PPARα) and increases the number of mitochondria. Meanwhile, citrulline activates the AMPK/PPARα pathway, amplifying the AMPK-mediated oxidative metabolism [14,43,44,54]. This collaboration collectively drives the formation of oxidative muscle fibers. Secondly, SH3KBP1 maintains sarcoplasmic reticulum calcium homeostasis, while glycerol-3-phosphate reinforces *SH3KBP1*-mediated calcium signaling by regulating myocyte membrane integrity [24], further stabilizing the oxidative phenotype of muscle fibers; *TRARG1* enhances glucose oxidative metabolism by promoting *GLUT4* translocation, while serine maintains mitochondrial redox homeostasis to reinforce the *TRARG1*-mediated energy production [43,52]. The Large White pigs exhibited dominant expression of fast muscle fiber-related genes (e.g., *MYH1*) centered on uridine 5′-monophosphate (UMP), which participates in pyrimidine metabolism to regulate cell proliferation and further accelerate the transformation of muscle fibers toward glycolytic types while weakening the activity of mitochondrial function-related metabolic pathways [51]. These data suggest that multiple levels of gene–metabolite interactions are important for the regulation of differential muscle fiber development among breeds.

## 5. Conclusions

This study systematically investigated muscle fiber composition, gene expression, and metabolic profiles in longissimus dorsi muscle of Huainan and Large White pigs during postnatal development. The myosin ATPase staining revealed significantly higher proportions of oxidative (type I) fibers in Huainan pigs. Transcriptomic analysis identified 18 differentially expressed genes that potentially maintain oxidative muscle fibers and meat quality. Metabolomic profiling showed increased levels of uridine 5′-monophosphate, carnosine, etc., in Huainan pigs, these metabolites enhance antioxidant capacity and energy supply to sustain oxidative fiber function. Integrated transcriptome–metabolome analysis revealed a synergistic regulatory network where oxidative metabolism genes (e.g., *KLF4*) and metabolites (e.g., citrulline) in Huainan pigs formed an AMPK-mediated “gene–metabolite” loop to promote mitochondrial function and fiber differentiation. Our findings provide multidimensional insights into muscle fiber regulation and support genetic improvement of indigenous pig breeds.

## Figures and Tables

**Table 1:** Primer information.

Gene	Primer Sequences (5′-3′)	Product Length/bp
*PPP1R15A*	F: TGAGGAGGAAGAGGATGGGG	95
	R: GATACATCTGGTCCCTGCGG	
*CCN1*	F: GTGAAGAAGTACCGGCCCAA	124
	R: CGTTCTTGGCAAACGTCTCC	
*IGF1R*	F: ACAGAGAACCACGAGTGCTG	97
	R: CGTAGTAGTAGTGGCGGCAG	
*SIX4*	F: GGGTCTACCTCCCAGGATGT	90
	R: AGTACTGGGGCTGTAGGAGG	
*MYOD1*	F: AACTGTTCCGACGGCATGAT	156
	R: CACGATGCTGGACAGACAGT	
*MSTN*	F: TGAGACCCGTCAAGACTCCT	123
	R: CAGTGCCTGGGTTCATGTCA	
*GAPDH*	F: ACCAGGTTGTGTCCTGTGAC	94
	R: AGCTTGACGAAGTGGTCGTT	

**Table 2:** Muscle fiber area of Huainan and Large White pigs at different developmental stages.

Breed	0 d	7 d	14 d	21 d
Huainan (um^2^)	96.45 ± 3.86	273.90 ± 8.91	362.90 ± 10.21	612.80 ± 15.43
Large White (um^2^)	74.47 ± 2.42	271.20 ± 8.28	376.30 ± 13.26	554.30 ± 19.74
*p*-value	0.86	0.83	0.99	0.00

Note: Values are presented as Mean ± SEM (n = 3).

## Data Availability

The original contributions presented in this study are included in the article/Supplementary Materials. Further inquiries can be directed to the corresponding authors.

## Supplementary Materials

The following supporting information can be downloaded at https://www.mdpi.com/article/10.3390/biology14101409/s1. Figure S1. Statistics of the number of differentially expressed genes within and between breeds. (A) The number of differentially expressed genes within each breed among consecutive developmental stages. (B) The overlap of differentially expressed genes between Huainan pigs and Large White pigs across four developmental stages (0, 7, 14, and 21 days old). Corresponding to Supplementary Table S4. Figure S2. PCA of genes in four stages of Huainan pig and Large White pig. (A) 0 days old; (B) 7 days old; (C) 14 days old; (D) 21 days old. Figure S3. KEGG enrichment analysis of differentially expressed genes between Huainan pigs and Large White pigs at different development stages. (A) Day 0; (B) Day 7; (C) Day 14; (D) Day 21. Table S1. Quality control results of transcriptome sequencing data. Table S2. Differentially expressed genes within each breed between consecutive developmental stage. Table S3. Differentially expressed genes between Huainan pigs and Large White pigs at four developmental stages. Table S4. Genes list for each section in the Venn diagram of Supplementary Figure S2A. Table S5. Information on the 18 shared differentially expressed genes between Huainan pigs and Large White pigs across the four stages. Table S6. Full GO enrichment of differentially expressed genes between Huainan pigs and Large White pigs at day 0. Table S7. Full GO enrichment of differentially expressed genes between Huainan pigs and Large White pigs at day 7. Table S8. Full GO enrichment of differentially expressed genes between Huainan pigs and Large White pigs at day 14. Table S9. Full GO enrichment of differentially expressed genes between Huainan pigs and Large White pigs at day 21. Table S10. Full KEGG enrichment of differentially expressed genes between Huainan pigs and Large White pigs at day 0. Table S11. Full KEGG enrichment of differentially expressed genes between Huainan pigs and Large White pigs at day 7. Table S12. Full KEGG enrichment of differentially expressed genes between Huainan pigs and Large White pigs at day 14. Table S13. Full KEGG enrichment of differentially expressed genes between Huainan pigs and Large White pigs at day 21. Table S14. Lists of differentially accumulated metabolites: intra-breed variations and inter-breed shared metabolites across four developmental stages.

## Abbreviations

The following abbreviations are used in this manuscript:

ATPase	Adenosine triphosphatase
DEGs	Differentially expressed genes
DHA	Docosahexaenoic acid
FPKM	Fragments per kilobase of exon model per million mapped fragments
GO	Gene ontology
HN pig	Huainan pig
KEGG	Kyoto Encyclopedia of Genes and Genomes
KLF4	KLF transcription factor 4
LW pig	Large White pigs
MTA	5′-Methylthioadenosine
MYH1	Myosin heavy chain 1
MYH4	Myosin heavy chain 4
MYH7	Myosin heavy chain 7
MyoD	Myogenic differentiation
NO	Nitric oxide
NOS1	Nitric oxide synthase 1
OPLS-DA	Orthogonal partial least squares discriminant analysis
PCA	Principal component analysis
PCs	Principal components
PGC-1α	PPARG coactivator 1 alpha
PPARα	Peroxisome proliferator activated receptor alpha
QC	Quality control
qRT-PCR	Quantitative real-time PCR
RSD	Relative standard deviation
SH3KBP1	SH3 domain containing kinase-binding protein 1
THRSP	Thyroid hormone responsive
TRARG1	Trafficking regulator of GLUT4 1
UMP	Uridine 5′-monophosphate
