# Supplementary material for: Multi-Omics Analysis and Comparison of the Developmental Characteristics of Muscle Fiber Types Between Huainan and Large White Pigs in Early Postnatal Period
Source: Biology (Basel). 2025 Oct 14;14(10):1409. doi: 10.3390/biology14101409 (PMC12562198; doi:10.3390/biology14101409)
Supplement: Supplementary file 1 [file biology-14-01409-s001.zip › Supplementary Figures-2025.9.24.pdf]

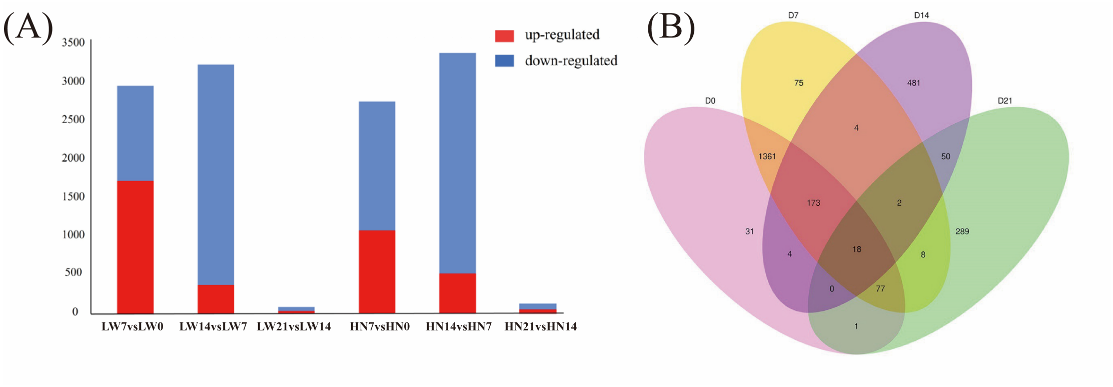

Supplementary Figure S1. Statistics of the number of differentially expressed genes within and between breeds.

(A)The number of differentially expressed genes within each breed among consecutive developmental stages. (B) The overlap of differentially expressed genes between Huainan pigs and Large White pigs across four developmental stages (0, 7, 14, and 21 days old). Corresponding to Supplementary Table S4.

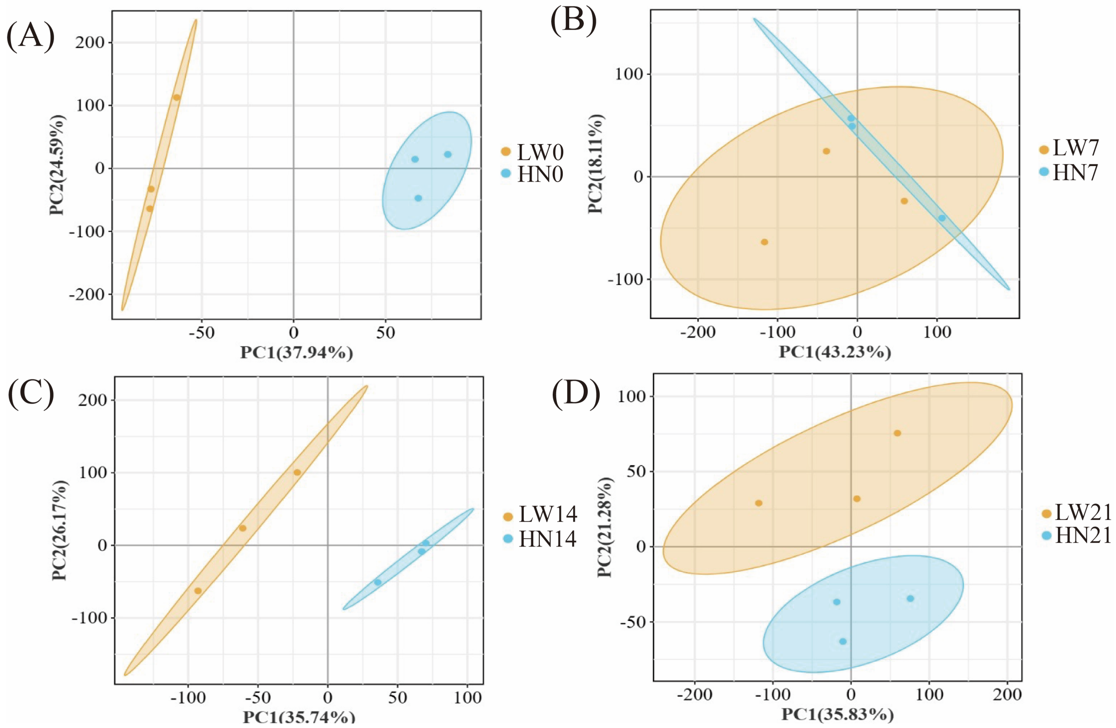

Supplementary Figure S2. PCA analysis of genes in four stages of Huainan pig and Large White pig. (A) 0 days old; (B) 7 days old; (C) 14 days old; (D) 21 days old.

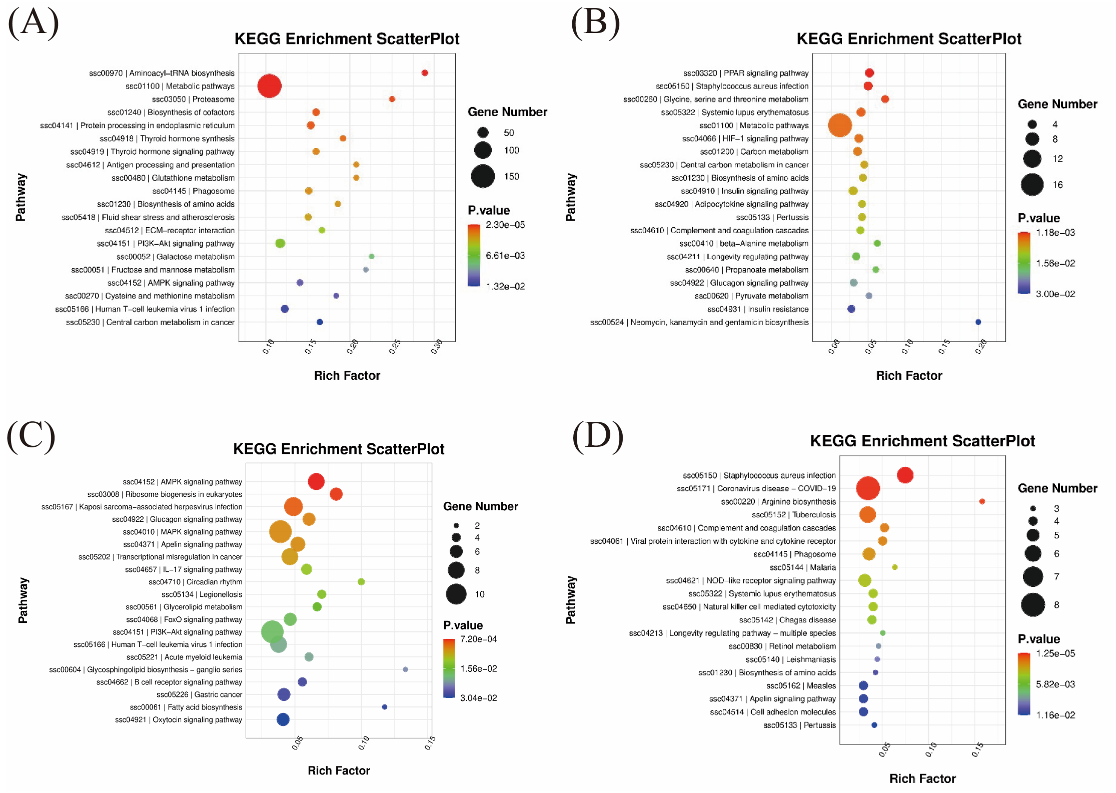

Supplementary Figure S3. KEGG enrichment analysis of differentially expressed genes between Huainan pigs and Large White pigs at different development stages. (A) Day 0; (B) Day 7; (C) Day 14; (D) Day 21.
